# Interdisciplinary Model for Scheduling Post-discharge Cardiopulmonary Care of Patients Following Severe and Critical SARS-CoV-2 (Coronavirus) Infection

**DOI:** 10.3389/fcvm.2020.00157

**Published:** 2020-08-14

**Authors:** Kristen Kopp, Michael Lichtenauer, Lukas Jaroslaw Motloch, Uta C. Hoppe, Alexander Egle, Helmut J. F. Salzer, Bernd Lamprecht, Josef Tomasits, Hannes M. Müller, Anna Dieplinger

**Affiliations:** ^1^Department of Internal Medicine II, Cardiology, Paracelsus Medical University, Salzburg, Austria; ^2^Department of Internal Medicine III, Hematology, Medical Oncology, Hemostaseology, Rheumatology and Infectious Diseases, Paracelsus Medical University, Salzburg, Austria; ^3^Department of Pulmonology, Kepler University Hospital, Linz, Austria; ^4^Medical Faculty, Johannes Kepler University, Linz, Austria; ^5^Institute for Medical and Chemical Laboratory Diagnostics, Kepler University Hospital, Linz, Austria; ^6^Department of Cardiac, Vascular, and Thoracic Surgery, Kepler University Hospital, Linz, Austria; ^7^Institute for Nursing and Practice, Paracelsus Medical University, Salzburg, Austria

**Keywords:** Covid-19, severe SARS-CoV-2 infection, post-discharge management, follow-up care, coronavirus

## Introduction

As Covid-19 can severely implicate the respiratory and cardiovascular systems, potential pulmonary, and/or cardiovascular sequelae may be anticipated in patients following severe and critical SARS-CoV-2 infection meriting coordinated post-discharge management to identify residual effects and to mitigate potential worsening of pre-existing conditions. According to current literature, 14% of patients with SARS- CoV-2 infection require hospitalization, of these, 5–14% have severe and 2–5% have critical manifestations of infection (1–4). While Covid-19 is known to primarily cause substantial respiratory pathology in hospitalized patients, such as pneumonia (75%) and acute respiratory distress syndrome (ARDS) (15%), it can also result in systemic complications affecting multiple organ systems including the cardiovascular system such as venous and arterial thromboembolic events (10–25%; 31–59% of ICU patients), myocardial injury (20–30%, >25% of critically ill; >55% in those with pre-existing CVD), cardiomyopathy (7–33% of critically ill), arrhythmias (17%, 44% of ICU patients), and cerebrovascular disease (up to 8%). Additionally, acute kidney injury (9%), hepatocellular injury (19%), hyperglycaemia and ketosis, ocular symptoms, and dermatologic complications have been reported (2, 4–7).

Although long-term outcomes of patients surviving severe SARS-CoV-2 infection are unknown, these patients have the potential to suffer substantial sequelae comparable to those in patients surviving ARDS, sepsis, and other acute illnesses. Survival from sepsis, for example, is associated with increased risks for mortality up to 2 years, new cognitive impairment, new physical disability, recurrent infections, and continued health deterioration (4). Long-term sequelae observed in survivors of severe ARDS during H1N1 influenza include significant exertion dyspnea, decreased diffusion capacity across the blood-gas barrier, as well as reduced quality of life including reduced exercise capacity, anxiety, depression, and/ or development of post-traumatic stress disorder (8).

Follow-up CT imaging at 4 weeks in patients with Severe Acute Respiratory syndrome (SARS) showed that one third of patients with persistent respiratory symptoms had findings of fibrosis, including interlobular and intralobular reticulation, traction bronchiectasis and, more seldomly, honeycombing (9). In another CT study of convalescing SARS patients 51 days after symptom start, follow-up CT showed air trapping (92%) ground-glass opacities GGO (90%) and reticulation (70%). While GGO and reticulation resolved by 5 months, air trapping caused by damage to ciliated respiratory epithelium persisted in 80% of patients (10). In Middle-East Respiratory Syndrome (MERS), 33% of patients showed evidence of lung fibrosis, affecting primarily the elderly, patients with prolonged ICU stays and those with greater lung involvement during the acute phase of infection (9).

With respect to cardiac sequelae following severe respiratory disease in recovered SARS patients, cardiac impairment was observed by echocardiography studies in short-term 30-days follow-up, especially in more critically ill patients (10). In the majority of patients with community-acquired pneumonia (CAP), cardiac injury was seen in 30-days follow-up, likely caused by myocardial oxygen supply and demand mismatch as well as an activated inflammation/coagulation system (11).

The European Society of Cardiology recognizes that SARS-CoV-2 infection has major implications on the cardiovascular system and that patients within the context of Covid-19 have increased risk of morbidity and mortality, especially those with established cardiovascular disease, common in patients with severe infection (12). Severe and critical SARS-CoV-2 infection is associated with acute myocardial injury, cardiac arrhythmias, likely caused by infection-induced myocarditis or ischemia, all with potential for new disease development. Following pneumonia, hypercoagulability, and systemic inflammatory activity can persist thus exposing patients to elevated long-term CV risk, justifying surveillance (12). An interdisciplinary model for scheduling follow-up care may serve as a practical tool for healthcare professionals to ensure that any infection-related sequelae following hospitalization for severe SARS-CoV-2 infection are identified and appropriately managed.

## Methodology

European Center for Disease Prevention and Control reports (ECDC), Center for Disease Control (CDC USA) and National Institutes of Health (NIH USA) reports, WHO Interim Guidance Reports, and current 2020 PubMed articles evaluating SARS-CoV-2 virus manifestations, diagnosis, severity, and discharge criteria of patients with confirmed Covid-19 were reviewed. PubMed Articles describing short and long-term outcomes in SARS, MERS, pneumonia, acute respiratory syndrome ARDS, and sepsis were evaluated.

## Rationale

The ECDC published a technical report in March 2020 comparing diverging international discharge and de-isolation criteria of patients hospitalized with Covid-19 found in national guidelines of Italy, China, Singapore, and the USA, and offered its own recommendations for discharge based on:

- Clinical criteria (e.g., no fever >3 days), improved respiratory symptoms, pulmonary imaging evidencing obvious absorption of inflammation, clinical assessment
- Laboratory evidence of SARS-CoV-2 clearance in respiratory samples, 2–4 negative RT- PCR tests for respiratory tract samples (nasopharynx and throat swabs with sampling interval ≥24 h) and if possible, serology with appearance of specific IgG (13).

With respect to post-discharge follow-up care, however, guidance is scant. The CDC China recommends that patients have follow-up visits 2 and 4 weeks after discharge, the National Centre for Infectious Diseases Singapore recommends clinic follow-up if indicated and daily wellness calls until day 14 after exposure, and the ECDC recommends 14 days of further isolation following discharge with regular health monitoring such as follow-up visits and phone calls, although specific guidance with respect to follow-up scope and content is not yet given (13).

The WHO report *Interim Guidance: Clinical Management of Covid 19*, released 27 May 2020 however anticipates potential sequelae in patients with severe and critical SARS-CoV-2 infection following treatment with mechanical ventilation, sedation, and/or prolonged bed rest based on evidence from general critical care populations. Post-intensive care syndrome (PICS) and severe respiratory illness may result in “a range of impairments including (but not limited to) physical deconditioning, reduced exercise tolerance, persisting fatigue, difficulties with activities of daily living, respiratory, swallow, cognitive, and mental health impairments” (14). According to WHO data, older people and patients of all ages with chronic diseases may be most susceptible to its impacts, including some patients recovering from severe COVID-19 who did not require admission to an ICU. The WHO recommends that patients must be referred for tailored inpatient, outpatient or community-based follow-up from post-acute to long term as indicated according to patient needs, with involvement of primary health care providers, relevant specialists, rehabilitation professionals, mental health, and psychosocial providers and social care services for coordinated care (14). A coordinated post-discharge care concept for patients surviving Covid-19 is therefore warranted to identify any cardiopulmonary sequelae and to mitigate possible worsening of preexisting disease following severe and critical SARS-Cov-2 infection. The literature has shown benefit of a well-structured transition phase to improve treatment outcomes and reduce readmission rates in management of other diseases such as heart failure, which may also develop in some patients following the infection (15–17).

While data examining residual effects after recovery from Covid-19 are still sparse, a number of sequelae especially affecting lung and heart function can be extrapolated from current literature. Initially defined by its pulmonary pathology and likely mediated via binding of SARS-CoV2 to ACE2 on lung epithelia, Covid-19 may have significant effects on long term-outcome with respect to pulmonary function. According to Shi et al., lung abnormalities such as bilateral ground-glass opacities progressing to or coexisting with consolidations were observable in CT imaging within 1–3 weeks of SARS-Cov-2 infection (18). Pulmonary fibrosis may occur due to scarring of the lung tissue, as was observed in SARS and MERS, potentially causing significant reduction in lung function and exercise capacity (19, 20), thus warranting follow-up in surviving Covid-19 target populations. Thoracic imaging with chest radiography (CRX) and computed tomography (CT) are key tools for pulmonary disease diagnosis and management (21). CT, however, is more sensitive for detecting parenchymal lung disease, disease progression, and alternate diagnoses. Therefore, in patients with reduced lung capacity and radiological signs of fibrosis at 1–2 months, continued follow-up according to ATS/ACCP guidelines will be required.

Recent publications have also shown direct endothelial cell involvement of vascular beds of different organs by the SARS-CoV-2 virus (22). This should be considered as a reason for cardiovascular events, endotheliitis of lung, heart, kidney, and liver, as well as liver cell necrosis. Covid-19 is characterized by coagulation activation with a high rate of venous and arterial thromboembolic events, including venous thromboembolism, pulmonary embolism, disseminated intravascular coagulation, or cardiovascular events (23). Coagulation testing is therefore warranted and subsequent therapy may be indicated.

Covid-19 may induce new cardiac pathologies and/or exacerbate underlying cardiovascular disease (24). Thus, cardiologists will aim to evaluate residual cardiovascular effects and myocardial injury following SARS-Cov-2 infection. Systemic inflammatory response coupled with localized vascular inflammation may lead to plaque rupture and activation of coagulation cascades, endangering patients for acute coronary syndromes (25). Heart failure may develop following myocarditis, sepsis, or multi-organ failure during infection, or may be caused by treatment side effects. Inflammation and ACE2 downregulation with ensuing endothelial dysfunction can translate into diastolic dysfunction, while hypoxemia may lead to right ventricular dysfunction indicative of myocardial injury. Thus, transthoracic echocardiography may be considered to evaluate left and right ventricular global function, any regional dysfunction, end-diastolic cavity dimensions as well as pericardial thickening or effusion (26). Additionally, cardiac MRI may better reflect structural pathologies of inflammatory myocardial damage. Cardiac arrhythmias, possibly caused by metabolic disarray, hypoxia, neuro-hormonal, or inflammatory stress, have also been associated with the infection (27) and if present will need follow-up evaluation. Cardiologists should be aware of the risk for development of chronic thromboembolic pulmonary hypertension in patients who experienced pulmonary embolism during infection (28). Follow-up is also an opportunity to address primary and secondary prevention strategies for cardiovascular risk control in all patients.

As the short, intermediate and long-term effects of Covid-19 are unknown, patients should be encouraged to participate in national and international registries or clinical studies to facilitate study of this disease. The monitoring of immune effects is also of particular importance.

## Post-Discharge Care Model

The proposed interdisciplinary model for scheduling post-discharge cardiopulmonary care of patients following SARS-Cov-2 infection (see Table 1) may serve as a practical guide for healthcare professionals to ensure that patients surviving severe and critical infection receive adequate cardiopulmonary follow-up care as we learn more about the residual and potentially chronic effects of the SARS-Cov-2 infection.

**Table 1:** Interdisciplinary model for scheduling post-discharge cardiopulmonary care following severe and critical SARS-CoV-2 infection.

Discharge	❖ **Scheduling for patients following severe or critical SARS-CoV-2 infection** ❖ **Scheduling for patients following moderate SARS-CoV-2 infection with exacerbation/worsening of preexisting comorbidities**
Medical professionals at discharge	• Schedule lab work (see parameters below) • Schedule chest CT scan for 1–2 months post discharge (unenhanced low dose CT), at index hospital if possible; chest x-ray (CXR) if CT not available • Schedule follow-up with **General Practitioner** at 1–2 months post-discharge • Evaluate, prescribe, discuss dischargemedications, any O_2_ use, care plan / appointments **with patient**, provide written instructions • If transfer to inpatient rehabilitation or long-term care facility, provide written instructions and care plan to managing physician • Involve **social worker**/**psychologist/nurse** where needed to address short, intermediate, long-term care/support (e.g., PTSD, psychological disorders, care provision) • Discuss potential participation and consent patient for any national/international registries or clinical trials and schedule appointments per protocol
**1–2 months post-discharge**	❖ **Diagnostic Testing, Care Coordination by GP/Internist**, ❖ **Referral and continued Follow-up when indicated**
Laboratory	• Complete Blood Count, C-Reactive Protein, LDH, AST/ALT, Urea, Glucose, Thrombin Time, Fibrinogen, Ferritin • Cardiac Biomarkers: CK, CK-MB, Troponin, NT-pBNP • For diabetes patients, also: Hemoglobin A1c • For Cancer patients, additional testing as instructed by managing oncologist
Radiologist	• Chest CT, or if not available, chest x-ray (CXR)
**General practitioner/internist**	• Clinical evaluation of symptoms (dyspnea, fatigue, psychological disorders) • Auscultation (determine signs of pulmonary fibrosis), • Oxygen saturation • ECG • Evaluation of laboratory, radiology and clinical findings, discussion with patient • Referral for further specialist examinations (e.g., **pulmonologist, cardiologist**) if indicated • Referral to **neurologist**, **nephrologist, endocrinologist** by suspicion of sequelae • Evaluate, prescribe, discuss discharge medications, any O_2_ use, and care plan with patient, provide written instructions • Involve social worker/psychologist if further support needed
Pulmonologist (if indicated)	• CT evaluation and discussion with patient • Physical exam: signs and symptoms • Lung function test • 6 min walk test • Blood-gas test • Reevaluation of medications, O_2_ use • Determine need for rehabilitation or intermediate/long-term care • Address primary/secondary prevention measures where applicable • Plan 6 and 12 month follow-up by any evidence of reduced functional capacity • Communicate findings and treatment plan to patient and general practitioner
Cardiologist (if indicated)	• Physical exam: signs and symptoms • ECG • Transthoracic echocardiography • Reevaluation/adjustments of medications • For patients with signs of heart failure: enrollment in heart failure program; for all HF patients: evaluate need for visiting heart failure nurse/rehabilitation program • For patients with arrhythmias, plan further evaluation (i.e., Holter monitoring, event recorder) • Address primary/secondary prevention measures where applicable • Schedule follow-up if appropriate • Communicate findings and treatment plan to patient and general practitioner
**After 2 months**	**Follow-up as needed and at the discretion of managing specialists**

*CT, computed tomography; CXR, chest X-ray; LDH, lactate acid dehydrogenase; AST, aspartate aminotransferase; ALT, alanine aminotransferase; CK, Creatinkinase; CK-MB, Creatinkinase-MB; NT-pBNP, N-terminal pro b-type Natriuretic Peptide; O_2_, oxygen; QoL, Quality of Life*.

**Target patient populations** for post-discharge Covid-19 follow-up care include:

- Patients who experienced severe illness^*^ defined as individuals who had respiratory frequency >30 breaths per minute, SpO_2_ < 94% on room air at sea level, a ratio of arterial partial pressure of oxygen to fraction of inspired oxygen (PaO_2_/FiO_2_) <300 mmHg, or lung infiltrates 50% (e.g., patients treated at an ICU requiring invasive ventilation or CPAP during SARS-CoV-2 infection)
- Patients who experienced critical illness^*^, defined as individuals who had respiratory failure, septic shock, and/or multiple organ dysfunction (e.g., patients treated at an ICU requiring ECMO during SARS-CoV-2 infection)
- Patients with chronic conditions (e.g., COPD, cardiomyopathy, coronary artery disease, cancer, chronic kidney disease, hepatic disease, and uncontrolled diabetes) in the presence of disease exacerbation or progression during/following moderate^*^, severe and critical SARS-CoV-2 infection, where moderate infection is defined as individuals with evidence of lower respiratory disease by clinical assessment, imaging and a saturation of oxygen (SpO_2_) > 94% on room air at sea level.
  ^*^ denotes NIH definitions of moderate, severe and critical illness (5).

As no guidelines on the timing of follow-up care for Covid-19 patients yet exist, this model schedules follow-up to occur at 1–2 months post-discharge based on several considerations. According to the previously cited radiological studies evaluating sequelae in patients following SARS and MERS infection, radiological follow-up was performed 1–2 months after start of infection (9, 10). In patients with confirmed pulmonary fibrosis, the American Thoracic Society recommends mid- to long-term follow up in 4–6-months intervals (29). With respect to cardiac involvement and the timing of follow-ups, the ACCF/AHA Guideline for the Management of Heart Failure was consulted with respect to recommendations for transition of care following hospitalization for acute cardiac decompensation. A follow-up visit within 7–14 days and/or a telephone follow-up within 3 days of discharge for acute cardiac decompensation is deemed a Class IIa recommendation (17). The 2016 *European Society of Cardiology Guidelines for the diagnosis and treatment of acute and chronic heart failure* detail the benefits of regular monitoring of heart failure patients, especially during periods of instability or for optimization of medications, noting benefits especially in older patients. Although timing of follow-up is not detailed, the ESC recommends provision of written action plans and prescheduling follow-up appointments shortly after discharge of patients with acute heart failure to reduce readmission rates (16). Therefore, scheduling transition care for Covid-19 patients potentially suffering from residual cardiopulmonary effects of the infection shortly after discharge is merited. The planning of post-discharge evaluations in the dynamic context of a pandemic, however, must be adapted with respect to post-discharge isolation recommendations, logistics, resource utilization, and health care system overburden. Thus, evaluation within 1–4 weeks post discharge at the height of a pandemic may not feasible for many patients. The model below suggests scheduling follow-up at 1–2 months, if not sooner, according to need and availability.

## Care Pathway

Hospital discharge personnel coordinate follow-up laboratory and radiological examinations, schedule a subsequent appointment with the patient's general practitioner or internist, and provide patient with written instructions. The patient's primary care physician or internist will serve as follow-up care coordinator. The interdisciplinary model provides guidance for specialist referral and testing dependent upon the patient's signs and symptoms, as well as radiological and laboratory findings. Due to the association of a more severe course of Covid-19 in those patients with underlying comorbidities, especially those with concomitant cardiovascular and pulmonary diseases, timely follow-up is imperative to identify any worsening of conditions and to initiate or adapt guideline-recommended therapies.

## Cost Analysis

We estimate that the costs per patient of the basic follow-up (Radiology, Lab, GP) to be € 1,026 according to the Austrian tariff system. In patients requiring specialist evaluation, an additional € 249 for pulmonary consultation and € 527 for cardiological consultation are estimated. However, cost-effectiveness cannot yet be determined until intermediate and long-term data become available for analysis.

## Conclusion

Short, intermediate and long-term effects following severe and critical SARS-CoV-2 infection are unknown, and significant sequelae may be expected, especially in patient populations experiencing ARDS, sepsis, and/or multiple organ dysfunction, as well as patients with exacerbation or progression of preexisting pulmonary or cardiovascular disease. Coordinated post-discharge management of Covid-19 patients is essential to identify and manage potential pulmonary or cardiovascular sequelae and mitigate worsening of pre-existing conditions following infection. This interdisciplinary model for scheduling follow-up care may serve as a practical tool for healthcare professionals to ensure that patients receive adequate treatment and post-discharge care following hospitalization for severe and critical SARS-CoV-2 infection.

## Author Contributions

KK wrote the manuscript. ML, LM, AE, HS, JT, and HM revised the manuscript. UH provided supervision. BL and AD provided supervision and revised the manuscript. All authors contributed to the article and approved the submitted version.

## Conflict of Interest

The authors declare that the research was conducted in the absence of any commercial or financial relationships that could be construed as a potential conflict of interest.
